# Supplementary material for: Serine 31 Phosphorylation-Driven Regulation of AGPase Activity: Potential Implications for Enhanced Starch Yields in Crops
Source: Int J Mol Sci. 2023 Oct 18;24(20):15283. doi: 10.3390/ijms242015283 (PMC10607544; doi:10.3390/ijms242015283)
Supplement: Supplementary file 1 [file ijms-24-15283-s001.zip › ijms-2641233-supplementary.pdf]

Supplementary Data

Serine 31 Phosphorylation-Driven Regulation of AGPase Activity: Potential Implications for Enhanced Starch Yield in Crops

Guowu Yu, Yuewei Mou, Noman Shoaib, Xuewu He, Lun Liu, Nishbah Mughal, Na Zhang and Yubi Huang

Corresponding author: Guowu Yu

State Key Laboratory of Crop Gene Exploration and Utilization in Southwest China, Sichuan Agricultural University, Chengdu 611130, China.

13862@sicau.edu.cn (G.Y.); Tel: 86-180-08039351

(A)

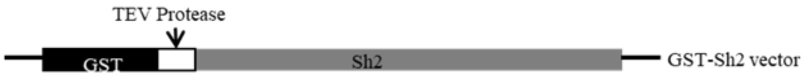

(B)

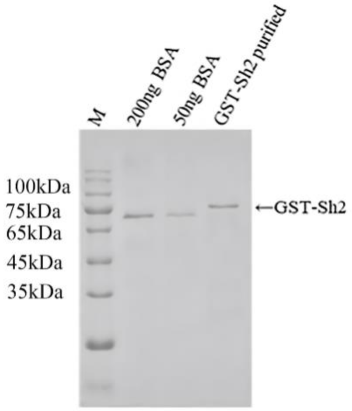

(C)

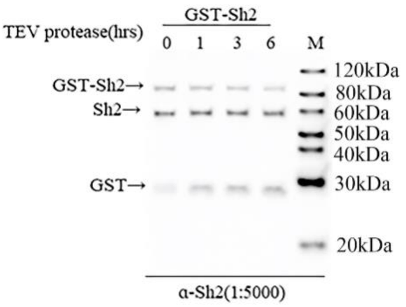

Figure S1. *In vitro* analysis of Sh2 antibody with a GST-purified antigen protein. (A) Schematic representation of the GST-Sh2 vector structure. The white section depicts GST tags and protein linkages at TEV protease sites. (B) Coomassie Brilliant Blue G-250 staining of purified GST-Sh2 protein and BSA standard, as depicted in the protein labeling diagram. (C) Western blotting of GST-Sh2 after TEV protease digestion for periods ranging from 1 to 6 hours. Each well contained 0.5μg of protein, and the antibody dilution was set at a ratio of 1:5000.

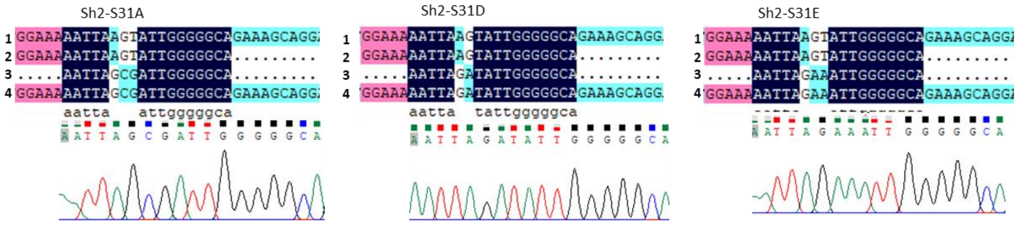

Figure S2. Sequencing results for Sh2 site mutations integrated into the puG-221 vector. (1) Represents the reference Sh2 sequence from the NCBI database. (2) Highlights the sequence region encompassing the Ser31 site. (3) Demonstrates the primers used for site-directed mutagenesis. (4) Provides the sequencing outcomes confirming successful mutations.
